# Using General Anesthesia plus Muscle Relaxant in a Patient with Spinal Muscular Atrophy Type IV: A Case Report

**DOI:** 10.1155/2011/743587

**Published:** 2011-10-19

**Authors:** Xiu-Fen Liu, Dong-Xin Wang, Daqing Ma

**Affiliations:** ^1^Department of Anesthesiology, Peking University First Hospital, Beijing 100034, China; ^2^Section of Anesthetics, Pain Medicine and Intensive Care, Department of Surgery and Oncology, Imperial College London, Chelsea and Westminster Hospital, London, UK

## Abstract

Spinal muscular atrophy (SMA) is a rare genetic disease characterized by degeneration of spinal cord motor neurons, which results in hypotonia and muscle weakness. Patients with type IV SMA often have onset of weakness from adulthood. Anesthetic management is often difficult in these patients as a result of muscle weakness and hypersensitivity to neuromuscular blocking agents as shown by (Lunn and Wang; 2008, Simic; 2008, and Cifuentes-Diaz et al.; 2002). Herein we report a case of anesthetic management of a patient with SMA type IV for mammectomy and review some other cases of SMA patients receiving different kinds of anesthesia.

## 1. Case Report

A 41-year-old woman (height 1.66 m; weight 62 kg) with SMA type IV was scheduled for mammectomy. The diagnosis of SMA type IV was based on clinical progressive symptoms of limb muscular weakness that began eight years ago. She has been on wheelchair since five years ago. At present, she even could not lift her arms up but had no difficulty in swallowing or breathing. She had a family history of SMA, her mother (died at 50 years old) and uncle both had the same disease. She had resection of right breast fibroadenoma under local anesthesia 6 years ago and right oophorocystectomy under epidural anesthesia 12 years ago. Physical examination revealed that she had a hoarse throat, normal gape degree, proximal muscular atrophy of the limb, muscular strength 2–4 degrees (with 4 degrees in the upper limb and 2 degrees in the lower limb), decreases of tendon reflex in extremities, no pyramidal tract signs, and paraesthesia. Chest X-ray showed increases in lung markings. ECG was normal. The laboratory examinations were normal except serum creatinine 31.00 *μ*mol/L (normal value is 44–133 *μ*mol/L). Bronchofiberscopy showed limitation of vocal fold abduction. A size 6 cm × 6 cm lump (benign fiber-epithelial tumor confirmed by a biopsy pathological examination) was found in her right breast, and at her request, her admission was scheduled to remove her right breast in fear of malignancy in the future.

On the operating day, no premedication was given to the patient. Electrocardiogram, cuffed blood pressure, and SpO_2_ were monitored. External jugular vein was inserted with an 18 ga catheter due to collapse of extremity veins. Anesthesia was induced with remifentanil (target-controlled infusion at plasma concentration of 3 ng/mL) and propofol (target-controlled infusion at plasma concentration of 3 *μ*g/mL) and a size 4 laryngeal mask airway (LMA) was tried to but failed. Then rocuronium 30 mg was given to facilitate the endotracheal intubation. The patient was intubated with an insertion of a size 7 endotracheal tube. Anesthesia was maintained with intravenous remifentanil (target-controlled infusion at plasma level of 2-3 ng/mL), propofol (target-controlled infusion at plasma level of 3-4 *μ*g/mL), and 50% nitrous oxide balanced with oxygen. The hemodynamic parameters were stable during the one-hour surgery. Morphine 3 mg was given intravenously 30 minutes before the end of operation. The patient woke up quickly after cessation of anesthesia and could raise her head. Antagonists of muscle relaxant (neostigmine 2 mg plus atropine 1 mg) were given. She was extubated and transferred to the surgical intensive care unit (SICU), where she recovered uneventfully. She returned to the general ward on the following day and was discharged from hospital 5 days after operation.

## 2. Discussion

SMA was first described by Guido Werdnig at the end of the nineteenth century and was divided into four types according to age at onset and severity. The sufferes of SMA type I and II are mainly infants and SMA type III are toddlers and teenagers. SMA type IV is a rare adult form with an incidence <0.5/10000 and is a milder form, which is inherited in either an autosomal dominant or rarely autosomal recessive manner. Patients with this type have a normal life expectancy, and motor impairment is mild without respiratory or nutritional problems [1–3].

SMA is characterized by degeneration of motor neurons of the spinal cord, which results in hypotonia, muscle weakness and atrophy (especially of the proximal muscles of the shoulder and pelvic girdle), and absence of deep tendon reflexes, with little or no impairment of sensory systems and the diaphragm and extraocular muscles remaining unaffected until the late stages of the disease. The diagnosis of SMA needs to be confirmed by electromyography and muscle biopsy. The pathology shows muscle degeneration, atrophic fibers with islands of group hypertrophy, and severe loss of motor neuron in the anterior horn region. Although there is no effective medical treatment for SMA at present, recent studies have elucidated the possible mechanisms of this disease development, and the therapeutic strategies have been identified and are in various stages of development [1–4].

The management of anesthesia in patients with SMA is often difficult because of muscle weakness, anesthesia-induced respiratory complications, hypersensitivity to nondepolarizing muscle relaxants, and succinylcholine-induced hyperkalemia. Also worries of neuraxial (epidural or spinal) blocks could worsen the weakness. Very little information is available in the anesthetic textbooks regarding the management of such cases although it was indicated that muscle relaxants, opioids, and thiopental could all have a prolonged duration of action [5, 6].

Several cases were reported about anesthetic management of patients with SMA without using muscle relaxants. Watts [6] reported a 25-year-old female with SMA type III presented for urgent corneal grafting due to keratoglobus under general anesthesia. Anesthesia was induced using alfentanil and propofol. After insertion of laryngeal mask airway (LMA), anesthesia was maintained with propofol and remifentanil infusions; controlled ventilation was maintained throughout the operation without muscle relaxants. Spontaneous respiration returned almost immediately the infusions that were stopped, and LMA was removed within 5 min. The author suggested that total intravenous anesthesia (TIVA) may provide an ideal anesthetic regimen for such patients. Habib et al. [7] reported a 23-year-old parturient of SMA type II undergoing elective cesarean section (CS) with general anesthesia (she had severe kyphoscoliosis and five Harrington rods inserted before she was ten). Anesthesia was induced with alfentanil and propofol, cricoid pressure was applied, and endotracheal tube was easily inserted without muscle relaxants. Anesthesia was maintained using 50% nitrous oxide in oxygen and 0.5–1% isoflurane; intraoperative analgesia was provided using morphine 6 mg given after delivery. The trachea was extubated at the end of the procedure, and the patient was transferred to the SICU. Following an overnight stay in the SICU and being moved to general labor ward, she was discharged 5 days later. The authors suggested that succinylcholine was classically used for rapid-sequence induction of anesthesia in the obstetric population. However, its use was contraindicated in patients with SMA because of the high risk of life-threatening hyperkalemia and rhabdomyolysis, and the use of nondepolarizing muscle relaxants needs also to be avoided. An increase of sensitivity to these drugs had been described in some lower motor neurone dysfunction caused by impaired production of choline accetyltransferase and acetylcholinesterase, and reduced concentration of acetylcholine at the endplate. An alternative method to achieve rapid-sequence induction without muscle relaxants was propofol-remifentanil technique. Kitson et al. [8] reported a 38-year-old type III SMA parturient receiving CS who had a history of tracheostomy during previous pregnancy. So she had a known failed intubation (hence the previous tracheostomy) and received awake fibreoptic intubation (FOI). Anesthesia was induced with alfentanil and propofol and maintained with isoflurane, nitrous oxide, and oxygen without muscle relaxants. She was extubated soon after the operation and placed onto a CPAP circuit. She recovered well and was discharged home 2 days later.

There also existed several case reports using muscle relaxants during general anesthesia. A 24-year-old female with type III SMA at 38 weeks' gestation was admitted for CS. Premedication included oral ranitidine 300 mg plus metoclopramide 10 mg. Anesthesia was induced with thiopentone and rocuronium; laryngoscopy and tracheal intubation proceeded uneventfully. Anesthesia was maintained by intermittent positive pressure ventilation with 50% nitrous oxide in oxygen with isoflurane 1%. With the exception of fentanyl 100 *μ*g administered after the delivery of the baby, no opiates were given intraoperatively. Reversal of neuromuscular blockage was not attempted until at least 40 min had passed from the administration of rocuronium. After neostigmine was given, spontaneous respiration quickly returned and anesthesia was discontinued. After regaining consciousness and despite adequate spontaneous respiration without dyspnea, there was marked residual weakness of the muscles of the upper limbs, head, and neck. The patient was transferred to SICU where she regained her preanesthetic pattern of muscle weakness after 8 h. She was transferred to the postnatal ward 24 h later and was discharged home one week later [9]. The author chose rocuronium for neuromuscular blockage as it allowed satisfactory intubating conditions after 60 s and had been used as an alternative to succinylcholine in obstetric anesthesia. Stucke and Stuth [10] first reported the use of a nondepolarizing neuromuscular blocking agent in an 18-month-old child with SMA (either mild type I or severe type II). Thiopental and alfentanil were used for anesthetic induction; a premature attempt at direct laryngoscopy provoked laryngospasm and inability to ventilate, so rapacuronium 9 mg (1 mg kg^−1^) was given. Within 60 s, ventilation by mask was restored and the child was intubated without difficulties. Within 15 min, the authors observed some diaphragmatic recovery, and after emergence from anesthesia, the child showed adequate respiratory effects but diminished strength of the upper extremity muscles. Small dose of midazolam was given to reduce the anxiety, and the patient was extubated within 5 h without any complications. They suggested that the pronounced difference in recovery times of the diaphragm and the upper limb muscles in the patient be most likely due to the different involvement of these muscle groups in the primary disease. 

In other cases, spinal, epidural, or combined spinal and epidural (CSE) anesthesia was selected for SMA patients during labor (either for CS or for labor analgesia). It was believed that parturients with SMA presented several problems for anesthesiologists. General anesthesia was complicated by underlying restrictive lung disease, sensitivity to nondepolarizing muscle relaxants, potential for hyperkalemia with succinylcholine and likelihood of difficult intubation. Regional anesthesia can be technically difficult. Epidural anesthesia may fail due to inadequate spread of local anesthetics, particularly if there had been corrective back surgery. Dose requirements for spinal anesthesia were difficult to predict, increasing the risk of either a failed or high block. CSE or continuous spinal technique may allow the block height to be titrated more carefully and should be considered ideal [11–15].

In our case, nondepolarizing muscle relaxant was used, and the time of onset and recovery of rocuronium remained in the normal range. The patient was extubated shortly after the operation and did not develop prolonged muscle weakness as in the other cases using muscle relaxants [9, 10]. The reason may be that these patients had different types of SMA, in which our patient had type IV SMA, with later onset and a milder impairment of motor function. In conclusion, nondepolarizing muscle relaxants could be used safely in SMA type IV patients, with the combined target-controlled infusion of remifentanil and propofol (TIVA), providing an ideal condition for anesthetic induction and maintenance. On considering recovery of muscle strength after anesthesia, it is advisable that train of four (TOF) monitoring should be carried out to secure the patients with SMA.
